# From 2D leg kinematics to 3D full-body biomechanics-the past, present and future of scientific analysis of maximal instep kick in soccer

**DOI:** 10.1186/1758-2555-3-23

**Published:** 2011-10-19

**Authors:** Gongbing Shan, Xiang Zhang

**Affiliations:** 1 Department of Kinesiology, University of Lethbridge, Canada; 2 Department of Physical Education, Xinzhou Teachers University, China

## Abstract

Biomechanics investigation on soccer kicking has a relatively long history, yet the body of knowledge is still small. This paper reviews articles published from 1960s to 2011, summarizing relevant findings, research trends and method development. It also discusses challenges faced by the field. The main aim of the paper is to promote soccer kicking studies through discussions on problem solving in the past, method development in the present, and possible research directions for the future.

## Introduction

The great attraction of soccer for millions of spectators may trace down to the basic idea of the game: goal-an idea that never ceases to fascinate. Various techniques for gaining goals are source of excitements, which make soccer the number 1 sport world-wide [1,2]. Currently, the game is played and watched on six continents with hundreds of millions of participants. While soccer is still keeping its strong trend in the traditional areas such as Europe and South Americans, the game has recently experienced significant growth in the other areas of the world with approximately 3 billion fans across every continent, i.e. almost half of the population on Earth is enjoying soccer as a sport [3].

The most significant change in the past two decades is that soccer is no more men's sport. Since the 2002 Women's World Cup, the popularity of women's soccer has grown tremendously in terms of both participation and as a spectator sport [4]. Various tournaments of international women's championships have promoted young females in North Americans, Europeans and Asians embracing the game, increasing the participants of this popular sport to a new high level. In fact, women will very soon represent 50% of the total North American soccer market [4].

However, scientific research on soccer and their performance variables are not proportional to its popularity. There are limited quantitative studies on soccer performance. Even the basic skills of kicking and passing are under-researched for the most popular, yet, still growing sport [5,6]. Majority of the previous biomechanical studies have been conducted in an attempt to understand the fundamental skills required by the sport-especially the maximal instep soccer kick [6-8]. Unfortunately, the number of scientific inquiries appears disproportionately low when compared to participation/scientific study ratios for some other sports, such as gymnastics. Therefore, the scientific understanding of this sport continues to lag behind its practice, with most participants acquiring skills through individual experience rather than research-based instruction. The aim of the article is to review and classify previous biomechanical studies on maximal instep soccer kick in order to show what have been done, what are the main problems that researchers are facing and where we might be heading in the future.

### Scope and Structure of the Review

The focus of this review paper is on biomechanical analyses, permitting one to draw conclusions on the characteristics/parameters, which can be used for reasoning the observed results. There are a plethora of further studies which are not included, for example: studies that are only descriptive in nature; studies concentrating on repeating previous investigations; and theoretical modelling and simulations for skill optimization.

**Figure 1:**
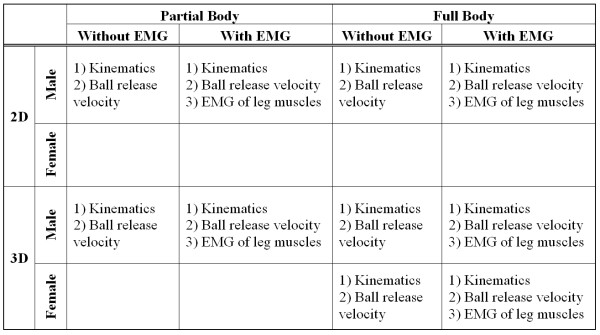
**Classification of previous studies in soccer kicking and their research goals**.

Throughout this review, studies are classified (1) by the spatial dimension (two-dimensional, three-dimensional), (2) by partial or full body, (3) by male or female and (4) by whether a motion analysis with/without EMG measurement. The motion analysis synchronized with EMG measurement can supply not only the spatial position of limbs and/or joints over a time period but also the related neural control (timing) and coordination among muscle groups. Such a synchronized system serves researchers with the information on how athletes prepare to produce a required action. Figure 1 shows the classification of previous studies and their research goals/purposes. As studies on female soccer start much later than those on males, only limited literatures are available, as such, no equivalent comparison in Figure 1 can be filled [4,5].

### 2D Kinematics Analysis

Success of a maximal instep soccer kick depends on multiple factors. Due to the nature of a team sport, the skill could be diverse and complicated in various situations, i.e. individual skills are influenced by the free flow of the play which makes the kicking context difficult to define for researchers. Further, it is difficult to collect unconstrained data due to limitations of both the laboratory and existing data gathering techniques. Therefore, researchers have necessarily made assumptions and simplifications in the hope of figuring out the main factors for training/improving the skill. One common test condition used so far is to kick stationary ball.

Literature showed that the scientific/biomechanical exploration of maximal instep kick using 2D film analysis began as early as 1960s [9]. With advance of motion capture technology, more 2D motion analyses were found between end of 1970s and end of 1990s [8,10-15]. These 2D motion captures varied at sampling rates ranging from 64 to 200 frame/s and focused on kinematic quantification of kicking leg. Therefore, the most kinematic data reported were results of partial body analysis in sagittal plane. Most often, the parameters used in these analyses were: ball speed, joint position changes, joint angle and angular velocity.

The major findings of 2D analysis can be summarized as follows. For performing an instep kick (last step of the kick), one should place the supporting foot at the side, and slightly behind, the ball. The kicking leg is first taken backwards swing with flexed knee. Then, in the following forward swing, the kicking leg should be carried out in a whip-like manner, i.e. forward rotation (both acceleration, and then, deceleration) should begin with the hip, followed by the rotations of knee and ankle. Such a timely control of the kicking leg is vital for the quality of instep kick [14]. Plagenhoef determined that, if the thigh quickly slowed down during the forward swing, the subsequent acceleration of the lower leg required less muscle power. Conversely, if less muscle force applied to the deceleration of the thigh, the leg extensors (e.g. quadriceps) had to increase the shortening speed in order to develop more power for achieving an equivalent foot kicking velocity. In this context, Plagenhoef indicated that improper swing control was also often the cause of muscle injury. It should be noted that the kicking leg movement is like an open mechanical chain, any change in a segment has an impact on the rest segments.

**Figure 2:**
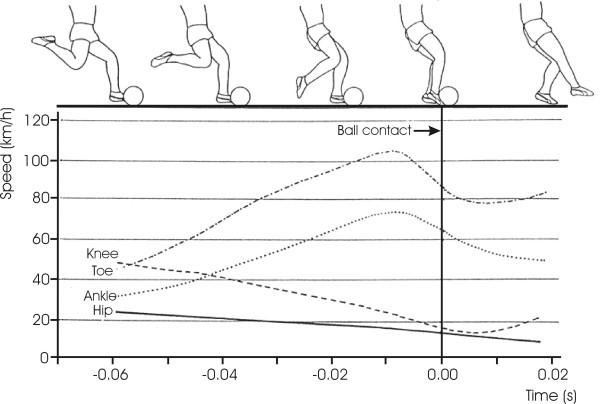
**Speed-time excursions of hip, knee, ankle and toe of a German national soccer player (modifies and translated from Kollath **[11]).

The whip-like movement of the kicking leg is also confirmed by a study done in Germany [11]. The study investigated the maximal instep kick using Bundesliga players (professional soccer players). In this study, the research investigated the acceleration and deceleration of thigh, shank and foot by examining the speed-time excursion of certain joints/points of the kicking leg (e.g. hip, knee, ankle and toe). The exemplary speed-time excursions of a national athlete are shown in Figure 2. It is apparent from the figure that shortly before the ball contact, the athlete slowed down the thigh while the speeds of ankle and toe increased dramatically, reaching their maxima at about 0.01 seconds before ball contact. Kollath suggested that such a control pattern (whip-like movement of the kicking leg) was auspicious for the momentum transfer from thigh to tibia, foot onto the ball.

**Figure 3:**
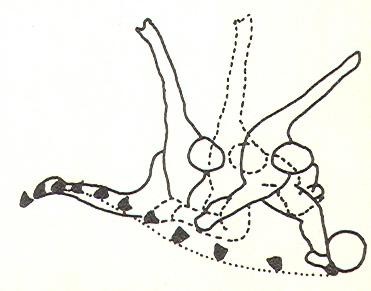
**Top view of the kicking foot movement during a maximal instep kick **[9].

Since most 2D analyses were done in the sagittal plane, the media-lateral movement of a kick cannot be revealed. A 2D study done by Roberts & Metcalfe remedied this deficiency [9]. Their analysis was done in the transverse plane. Their study made it clear that the path of the kicking foot was like S-curve to the ball, not as it was often assumed-a straight line (Figure 3). The authors concluded that approaching a ball with certain angle would benefit hip rotation, which was important for the lateral foot movement during a kick.

The other significant results from previous 2D analyses are the quantification of the maximum speed of the kicking foot as well as the ball release speed. There are various studies using different level athletes. Generally speaking, the maximal speeds of kicking foot are 25 km/h and more than 100 km/h for advanced players and elite athletes respectively. Consequently, the ball release speed ranges from 30 km/h to more than 120 km/h [8,9,11]. Although possessing scientific merit, the results of previous studies have limited practical use, i.e. athletes and coaches typically have no means to collect and interpret such data during training. In order to bridge the gap, some researches had tried to develop user-friendly evaluation methods. Their research goals focused on simplifying evaluation by measuring one or two parameters that conveyed the kick quality. Examples are:

(1) $${\text{V}}_{ball}=1.23\times {\text{V}}_{foot}+2.72$$

Where V = velocity of ball and foot, respectively [15].

(2) $$V_{ball}=\frac{V_{foot}\times M\times \left(1+e\right)}{M+m}$$

Where V = velocity of ball and foot, respectively, M = effective striking mass of the leg; m = mass of the ball; and e = coefficient of restitution [16,17].

Even so, these simplified models are not sufficiently "practical", since practitioners have to determine foot velocity by using motion capture/tracking technology.

In terms of injury prevention, one risk factor found among inexperience players is the contact location between foot and ball. While the ball is hit correctly by professionals (dorsal arch area of the foot), the amateurs or, sometimes, advanced players meet the ball more with the front part of the foot [18]. Such kicks lead often to an injury caused by rapid over-extension of the foot and result also in a reduced ball speed. It is recommended that training should begin with a slow leg swing, concentrating on kick accuracy. Only when there is a high degree of accuracy achieved, training can forward with an increased swing leg motion [10,18].

Finally, 2D analysis has also proved that the maximal angle between thighs of the last step or the movement amplitude of the last step is highly correlated to the ball release speed. A study done by Stoner & Bensira showed that there is significant difference existed between kicks resulting in 25 m and 45 m of ball's flight [19]. 45-meter kicks have greater movement amplitude at the hip as well as the last step length than those of 25-meter kicks. However, there is no differences existed in knee extension between both kicks. The result suggests that the range of motion of hips and/or the last stride length can be used to evaluate the training progress (or training effect).

In summary, 2D motion analysis has revealed certain insight related to maximal instep kick. There is no doubt that 2D studies have laid a foundation for quantifying the skill. However, it remains questionable whether 2D analysis can describe full-body movement without losing important characteristics. Clearly, to answer this question, further studies using other technologies, such as EMG and 3D motion capture are needed.

### Muscle activity studies using EMG measurement

For researchers, surface EMG seems to be a seductive technology because it provides a non-invasive means to acquire in-vivo data about physiological processes that cause muscles to produce a movement. It captures muscle activity during any movement of a body by measuring seizure potentials released by the muscles when they are activated. Consequently, measured potentials are typically used to fractionate a body's response process as well as get timing of neural control and coordination among muscle groups. Such analyses permit assessments of technical skills which, in turn, provide foundational information for motor skill analysis. Several studies employed EMG measurement in an attempt to characterize activation, coordination and intensity of selected muscles during soccer kicking [20-22]. The typical goals of these EMG studies were 1) to describe the activity of selected muscles for skill optimization and learning/training, and 2) to determine loading intensity during performance for injury prevention. But, there is a concern of using EMG measurement alone in an application, as it lacks the connection between body/limbs' movement and neural muscle contractions. Therefore, some researchers have synchronized EMG data with 2D motion analysis in order to link muscle activities to joint kinematics for more accurate analyses [21,23].

EMG quantification of leg muscles' activation and coordination should be a means to confirm the whip-like movement during kicking observed in 2D motion analysis. Theoretically, if leg muscles' control is in an optimized way, i.e. agonists and antagonists work with each others (do not against each others), EMG readings of selected muscles should reflect significant changes depending on the acceleration or deceleration of hip, knee and ankle. A study done by Shan et al was able to show that the quadriceps' activity of experienced soccer players did help perform a whip-like kick [4,5]. The result was obtained by comparing advanced athletes to amateurs. However, not all results of previous studies could confirm the whip-like movement in soccer kicking. The study done by Dörge et al showed that M. iliopsoas was active during the entire kicking motion and angular deceleration of the thigh segment did not increase the angular velocity of the shank [21]. The seemly contradictory results in previous studies prove actually the effectiveness of EMG in skill analysis. Whip-like movement is an optimized control state, resulting from years' training. As such, the higher an athlete's skill is (e.g. the German national player in Kollath's study [11]), the more obvious the phenomenon is. The subjects in Dörge's study may not reach their optimized states. Hence, EMG can be used as a tool for biofeedback training in practice.

Synchronized with motion capture, EMG data can been used as a supplementary information for loading analysis during a kick. Such quantification provides a basis not only for improving soccer kicking performance but also for injury prevention. In terms of EMG application in this regards, Brophy's study is a representative [23]. By investigating muscle activity changes of iliacus, gluteus maximus, gluteus medius, vastus lateralis, vastus medialis, hamstrings, gastrocnemius, hip adductors and tibialis anterior, the research found that significant interaction existed between the hamstrings and the tibialis anterior. Additionally, as expected, higher muscle loading was found in kicking leg, as the activation of iliacus, vastus medialis, gastrocnemius and hip adductors was significantly larger than those in non-kicking side. Further, the researchers compared the EMG of instep kick to that of side-foot kick and discovered that there were significant differences between the two types of soccer kick. The study suggested that injury prevention strategies should be linked to various techniques.

Finally, EMG reading can also be used to reflect a kick direction or accuracy. It is known that one of the EMG uses is to quantify the neural-muscular control or coordination among muscles involved in a movement, EMG data should demonstrate dissimilarity between kicks intending to different targets. The study done by Scurr and his colleagues did prove that EMG reading of the same muscle varied depending on target selection [24]. The results showed that kicks to the right targets produced significantly greater muscle activity than those towards the left targets. Additionally, kicks towards the top-right target had significantly greater muscle activity than that towards the top-left. Among all the conditions tested, kicking to the top-right corner of the goal demonstrated the highest quadriceps EMG level than those towards the rest corners. This result suggested that a top-right instep kick would be the most powerful kick for accelerating the ball; therefore, using this kick in a game would leave less time for goalkeepers to react.

Again, like 2D motion analysis, EMG (even synchronized with 2D motion capture) could only divulge certain aspects related to maximal instep soccer kick. Especially, most of the previous studies focused on lower limbs' movement and/or muscles' activities during kicking, a holistic picture of the body control as well as the upper body function remained still unknown to researchers and practitioners.

### 3D Analysis-From Partial Body to Full-Body & Multi-dimensional Data Analysis

There were a few studies address a 3D perspective of the soccer kick before 2000 [6]. The few existing 3D studies at that time employed also partial body model to explore or examine aspects that could be decisive for soccer kicking. The research of Rodano & Tavana was representative [25]. The study used opto-electronic system (100 Hz) for recording marker positions placed on the leg joints. Unfortunately, the results did not bring significant new aspects in comparing to the previous 2D studies. The influence of 3D analysis (or extension from 2D to 3D) was heavily limited, because the early 3D investigations still followed the partial body model, established in 2D analysis. However, empirical data suggested that upper-body movement was a necessary part of the skill control [6]. Failure to address the upper body movement during kicking would lead to an incomplete understanding of joint coordination and motor control for kicking skill. It is known that the main reason for neglecting upper body movement is technique difficulty, i.e. it is not easy to collect unconstrained data using full-body model in 3D space. But, for advancing knowledge, full-body analysis has to be the next step.

**Figure 4:**
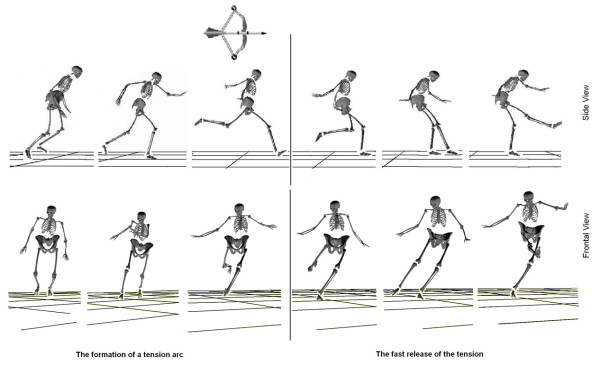
**The main feature of a maximal instep kick-a process consisting of tension arc formation and its fast release**.

The first full-body, 3D analysis of maximal instep kick was done by Shan and his colleague [6]. The study was done in a lab environment with the following settings: 1) ground conditions were made to mimic the effects of grass using a 2 cm thick wrestling mat, as such athletes could wear soccer shoes; 2) the kicking was towards a large, vertically-hanging wrestling mat (5 × 2 m^2^) with good impact-absorbing for reducing rebound. The setting, especially the large size of the mat (an easy target), made the subjects able to kick the ball as hard as they could without fear of any consequences. A nine-camera VICON motion capture system (120 frames/s) was used to capture full body kinematics by tracking 45 reflective markers. 42 markers were used to define a 15-segment model, representing the full body and 3 markers were used to determine the ball's movement. One advantage of such a test environment was to permit considerable freedom of movement for the subject without negatively influencing their motor control. Therefore, in the study, researchers placed no restriction on the subjects' movements, i.e. subjects executed their movements in their usual "style". The results revealed that the key characteristics of maximal instep kick could be extracted as the formation of a tension arc, and the fast release of this tension arc-a quasi whip-like leg movement with complimentary upper body movement (Figure 4). The formation of the dynamic tension arc involved: 1) kick-side hip over-extension and knee flexion, 2) trunk twist towards non-kick-side, and 3) non-kick-side shoulder extension and abduction (Figure 4, left). The release of the arc consisted of: 1) quasi whip-like control sequence of the kicking leg, and 2) upper trunk flexion and twist towards kick-side, and 3) the non-kick-side shoulder flexion and adduction during kick (Figure 4, right). Further, by comparing kicking characteristics of advanced players with that of novices, the researchers confirmed that the tension-arc created a better condition for generating an explosive muscle contraction through muscle pre-lengthening and made the kick more powerful. As such, for evaluating a kick quality, the researchers concluded that the maximal distance between the kick-side hip and the non-kick-side shoulder provided a quantitative means of judging the kick quality. These results indicate that full-body 3D analysis is more appropriate for supplying insight than 2D analysis for kick skill analysis.

As stated in 2D analysis, synchronizing motion capture with EMG presents a promising way to measure, analyze and understand any complex skill of human motor skill. Only, for soccer kicking analysis, wired EMG would put constrains on kicking, negatively influencing data collection. One solution would be to use wireless or telemetry EMG. Such system reduces the movement constrains to a test person tremendously, and allows researchers to obtain data from an un-manipulated process. Therefore, the analysis based on such a synchronized system will reduce un-wished compound influence and reveal the nature of a movement. This kind of data collection technology was applied in the further studies by Shan and his colleagues for female soccer kicking investigation [4].

Women soccer has been booming for the past two decades. However, scientific research on female soccer players is still rare [4]. For training basic skills, such as an instep kick, it is important to know the characteristics of an optimized style, as the knowledge will help coaches to develop goal-oriented drills which might aid in speeding up the learning process. One way for achieving this goal is to quantitatively determine the characteristics of advanced players as well as the novices, so that coaches can design their training programs based quantitatively determined factors of skill performance. Shan's study measured two groups of female subjects; a novice group (N = 10) and a skilled group (N = 10) using a 9 high-speed cameras (120 frame/s) system and a wireless EMG (1080 Hz). The main findings of the study included: 1) Upper body movement contributed significantly to skill effectiveness. Skilled players utilized more trunk twist and arm extension as well as abduction on the non-kick-side during ball contact phase. Such upper body control was hardly noticeable in novice subjects. The results further confirmed that full-body analysis would be the only way to obtain these differences between skilled and novice players, i.e. such characteristics can not be revealed by 2D motion analysis. 2) Skilled subjects demonstrated significantly greater range of motion in both the hip and the knee joints, leading to higher velocity of kick foot prior to ball contact. On the contrary, novice kicker used the knee as primary source of inertia for increasing kick foot velocity. And 3) Revealing by the EMG data, skilled players employed muscle control in a way that produced an 'explosive' pattern of muscle activation which contributed to an increase of ball release velocity. Such a control pattern could be characterized by: greater final intensity in muscle contraction, more rapid acquisition of maximal contractions, and more accurate timing of the gross movement pattern. In summary, experienced female players utilize greater control in multiple segment sequencing, including the trunk and upper extremity and superior timing of muscular contractions in order to maximize power in the kick. While, novices try to perform a kick as more simplistic as possible (e.g. mainly control the knee), i.e., the novice kick is comprised of movements that are noncomplex and easy to control.

### Gender Influence & Injury Risk

In contrast to men's soccer, women's soccer has received minimal research attention. This lack of interest on researching women's soccer has the cost of leaving a vacuum to be filled by men's experiences [5]. Most often, results obtained using male athletes are transferred to female ones in training without considering the gender differences including anthropometrical structure, body flexibility, muscle force and power. As such, the contrast of gender differences in soccer skills is highly desired by practitioners. The series studies by Shan and his colleagues remedied this deficit [4-6]. These studies were to quantitatively describe the characteristics of male & female athletes' kicking, and to compare them in order to determine the gender influences on maximal instep soccer kick.

**Figure 5:**
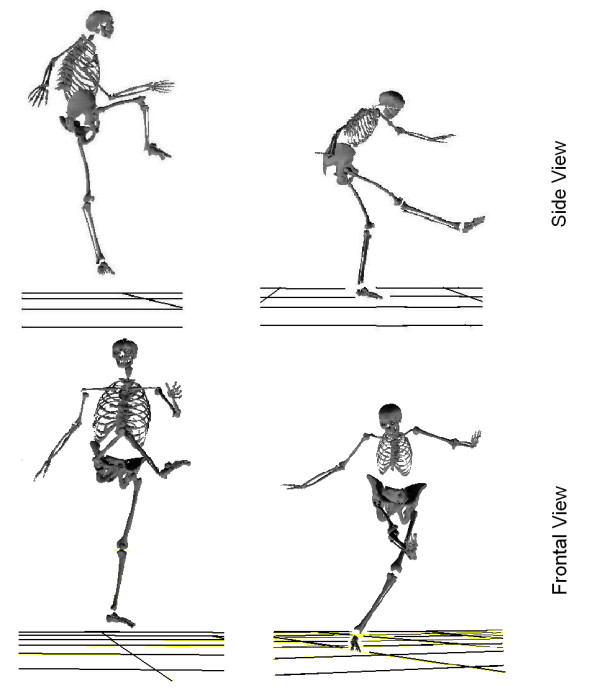
**Gender comparison of maximal instep soccer kick of advanced athletes-posture after ball contact (left-male, right-female)**.

The relevant findings include: 1) after a powerful kick, males naturally follow through with a jump in order to dissipate residual leg momentum while females avoid this airborne phase; instead they counteract the momentum with upper body flexions (Figure 5). The reasons for this discrepancy are unknown. However, the researchers speculated that vibrations caused by jump take-off and landing could be eliminated by the female technique. Thus it is possible to explain that the gender-based difference could be caused by anthropometry, e.g. the male body has less chest mass (as well, more solid) than the female body, therefore it is less prone to discomfort caused by vibrations from jumps. Unfortunately, there is still no written record of different training processes based on gender [5]. Therefore, it is an interesting topic for future studies on how female players develop this technique during training and determine if it is unconsciously developed by the body for self protection. 2) The male and female players have significant difference in ROM (range of motion) of trunk flexion during kick. In order to counteract residual leg momentum without being airborne, it becomes necessary for females to utilize a significantly larger trunk flexion. This then alters the whole body control. This could be a reason for the significant difference of larger run-up angle between males and females. 3) A difference exists in the degree of tension arc formed between gender groups. Male players displayed more muscle pre-lengthening for quadriceps, rectus abdominis, pectoralis major, obliquus externus abbominis and obliquus internus abbominis, thus creating a tighter tension arc; it should then conclude that more muscle power is generated by the male kick. This is in agreement with the higher ball release speed found in male group. 4) Male skeletal muscles generally contract faster and have higher maximum power output than female muscles [26]. However, the data showed that training can alter this situation. The advanced female players demonstrated greater use of the quadriceps and with more efficiency (e.g. higher EMG maximum and faster increase rate of muscle tension) than that of inexperienced male players; as such their muscles can generate more power than novice male during a kick.

### Limitations and Future Directions

Soccer is a team sport, and individual skill is only one of many vital factors that can define success. There is variety of soccer skills; however, maximal instep kick is the main offensive action during the game. Improvement of the skill is one of the most important aims of training programs in young players. Practically, it is difficult to collect unconstrained data due to limitations of both the laboratory and existing data gathering techniques. In order to overcome these difficulties, researchers have necessarily made assumptions and simplifications, these simplifications bring limits to the research results. One of the considerable limitations is that the studies so far deal with the kick using stationary ball. It is no doubt that it is the first step. Moving to the next level-studying kicks using moving ball-is a great challenge for researchers.

Although possessing scientific merit, previous studies (both 2D and 3D) have little impact on practitioners, because the results have limited practical use. Athletes and coaches typically have no means to collect, evaluate and interpret such data under typical training conditions, i.e. there is a gap between scientific study and practical application. For example, the maximal distance between the non-kick-side shoulder and the kick-side hip during kick is highly correlated to kick quality, as such, the parameter can be used to evaluate training effect [6]. However, there is no easy way other than using 3D full-body analysis to obtain the parameter. Obviously, practitioners can hardly use this parameter during training. Another example, some researchers have tried to develop user-friendly evaluation methods, including evaluations by measuring one or two parameters that conveyed the kick quality/power, for example, using regression equation of V_ball _= 1.23 × V_foot _+ 2.72 [15]. Such evaluation still needs to measure velocity of kicking foot, i.e. not sufficiently "practical", as they use human resource-intensive motion analysis technology.

For overcoming the current limitations, future studies could follow two possible paths: 1) developing handy measuring devices based on the results of previous studies and 2) exploring parameters which are easily obtainable in the field during training. For the first case, a carry-on device could be developed for athletes to wear during trainings. The function of the device is to supply the changing range of distance between the non-kick-side shoulder and the kick-side hip during kicking. Such a biofeedback would help both athletes and coaches in evaluating training effects. For the second case, one possibility would be to determine the regression equation(s) between last stride length and ball release velocity in future investigation. Previous studies showed that 1) longer-distance kickers had greater movement amplitude at the hip as well as the last step length than shorter-distance kickers [19]. From practical point of view, it is more difficulty for soccer coaches and players to measure kicking foot velocity during training than to measure last stride length. The latter is simply a distance measurement of footprints left on the soccer field. Summarized above, increasing applicability should be emphasized in future studies. The results obtained through such studies have great potential to bridge the gap between researchers and practitioners, as such, make soccer studies more meaningful to coaches and athletes.

Finally, future exploration could also focus on the development of new training programs based on results of current studies, for example, female training course should be developed based on the gender differences. Or, it is interesting to investigate how female players develop different technique from males during training; if there is any mechanism existed, e.g. unconsciously developed by the body for self protection. The answers will definitely contribute to teaching and learning of soccer kicking skill.

## Conclusion

Biomechanics study on soccer kicking has relative long history, beginning in 1960s. It experienced from partial-body, 2D film analysis to full-body, 3D analysis using synchronized data collection system. Collectively, the results show that an effective soccer kicking utilizes the entire body and a complex multi-joint coordination to generate maximal impulse to the ball. We can abstract the maximal instep kick as the formation of a tension arc, and its fast release using a whip-like leg movement. During the process, an increase in certain muscles' pre-lengthening can be observed. The pre-lengthening contributes to an explosively powerful kicking. Further, it is found that through training, male and female athletes have developed different techniques, characterized by run-up angle, trunk flexion and the dynamic posture after ball contact. Finally, during training, practitioners should pay special attention to repetitive injuries in muscles like the Adductor Magnus. Although soccer kicking studies have revealed certain insight, scientific investigation so far has little impact on practitioners. The main reason is that the researches have not reached practical level. In order to bridge the gap between scientific research and application, future studies should aim at developing user-friendly methods for evaluating training effect and improving learning efficiency for soccer athletes and coachers.

## Competing interests

The authors declare that they have no competing interests.

## Authors' contributions

Both authors selected the references, discussed the paper structure and co-wrote the paper during manuscript preparation. They have read and approved the final manuscript.
